# Completion of a standardizable competency-based research training program improves understanding and preparedness for both new and experienced clinical research professionals

**DOI:** 10.1017/cts.2024.690

**Published:** 2024-12-26

**Authors:** Lucy Palmer, Sandra E. Morones, Heidi T. Jacobe, Rhonda Oilepo

**Affiliations:** 1 Office of the Provost, Office of Clinical Research, University of Texas Southwestern Medical Center, Dallas, TX, USA; 2 Department of Dermatology, University of Texas Southwestern Medical Center, Dallas, TX, USA; 3 Human Research Protection Program, University of Texas Southwestern Medical Center, Dallas, TX, USA

**Keywords:** Research personnel, education program, curriculum development, clinical research, workforce development

## Abstract

Clinical research professionals (CRPs) are integral to the academic medical center workforce, research operations, and daily clinical research tasks; however, due to inconsistent training, there is a shortage of qualified CRPs. The Joint Task Force for Clinical Trial Competency created a competency framework for CRPs, which has demonstrated positive results from various institutions, but training programs have been limited in standardization, replicability, and dissemination. To improve this, we designed the University of Texas Southwestern (UTSW) Medical Center Clinical Research Foundations (CRF) training program, which is a competency-based online self-paced CRP training curriculum hosted via the Collaborative Institutional Training Initiative (CITI) portal. We examined feasibility, acceptability, and uptake of the UTSW CRF training on an institutional scale and were pleased to find this curriculum is not only feasible but has high levels of acceptability. Furthermore, faculty, clinicians, and trainees voluntarily completed this training program indicating utility across diverse groups. The UTSW CRF combines the existing CITI training modules with UTSW-created material, providing an optimal balance between generalized clinical research education and institutionally tailored content. We believe the UTSW CRF curriculum could serve as a plug-and-play foundational model for other research centers to tailor according to their audience and institutional needs.

## Introduction

Clinical research professionals (CRPs) are integral members of the academic medical center workforce and critical to research operations. CRPs are responsible for the majority of clinical research tasks, including protocol and budget development, patient recruitment and enrollment, regulatory submissions, and data management processes. CRPs have a variety of job titles, each with different education and training requirements. Despite having responsibilities that are frequently characterized by large workloads and complex tasks, CRPs typically have inconsistent training, as well as limited opportunity for career progression and advancement [1]. Prior reviews have identified additional barriers to recruiting and retaining CRPs, including a lack of institutional resources, gaps in training, and minimal professional development possibilities [2]. Taken together, this environment contributes to a shortage of qualified CRPs, leaving positions unfilled with high rates of turnover [3]. The crisis in CRP staffing has implications for ongoing research development, cost, and safety [4]. As a result, the National Institutes of Health (NIH) has emphasized the importance of adequate support and training for CRPs to ensure the safe and effective delivery of clinical research [1].

Despite the need for CRP support and training, standardized, high-quality instruction across and within institutions is lacking [5], resulting in inconsistent and ineffective onboarding, guidance, and professional development [6]. The absence of structured support and training creates a barrier to CRP professionalism, which may trigger a lack of personal confidence and professional identity-related apprehension [7]. CRP professionalism requires a focus on core competencies, which are part of the CRP job description. In 2014, the Joint Task Force (JTF) for Clinical Trial Competency created a competency-based framework for CRPs covering eight essential clinical research domains [8] with leveled objectives, resulting in 47 Leveled Competency Statements [9]. These competencies have been continuously refined over the last 10 years, as several institutions have utilized this framework when developing their training initiatives [10−12]. Early indications from institutions who have integrated this framework are positive [13−16], but current training offerings are limited in terms of standardization, replicability, and potential for dissemination. These efforts are further hindered by cost, as they require upfront investment from the institution for training material development and ongoing funds for instructional support. Moreover, most institutions that have invested in competency-based frameworks have had limited insight into whether the needs of their participants were actually met [2,6].

To address these barriers, we designed and implemented a novel competency-based online CRP training curriculum, called the University of Texas Southwestern (UTSW) Medical Center Clinical Research Foundations (CRF) training program. We examined the feasibility, acceptability, and uptake of the UTSW CRF on an institutional scale. This training initiative is a self-paced CRP onboarding program, which can be adopted across institutions to provide standardized CRP training and professional development, as well as site-specific customization.

## Methods

These activities were reviewed by the UTSW Human Research Protection Program and determined to be part of program evaluation. Therefore, the subject matter of this paper was deemed non-regulated research, so no Institutional Review Board approval or oversight was required.

### Overview

In order to address institutional barriers to recruitment and retention of CRPs, a team from the UTSW Office of Clinical Research (OCR), Human Research Protection Program, and Clinical Translational Science Award determined that a centralized approach to training through the OCR was a vital requirement to successful education of new research staff. In collaboration with Human Resources, a list of clinical research job titles was identified (Supplemental Material 1), and we agreed that from July 2023, all new hires starting in these roles would be required to complete the CRF training as part of their institution-wide onboarding. Individuals transitioning from an existing approved clinical research job title at UTSW were exempt from this requirement, as determined by the OCR. Course completion was required within the first 30 days of hire, and individuals were held accountable to completion by the OCR with potential withdrawal of research privileges for noncompliance.

### Program Design

**Figure 1.**
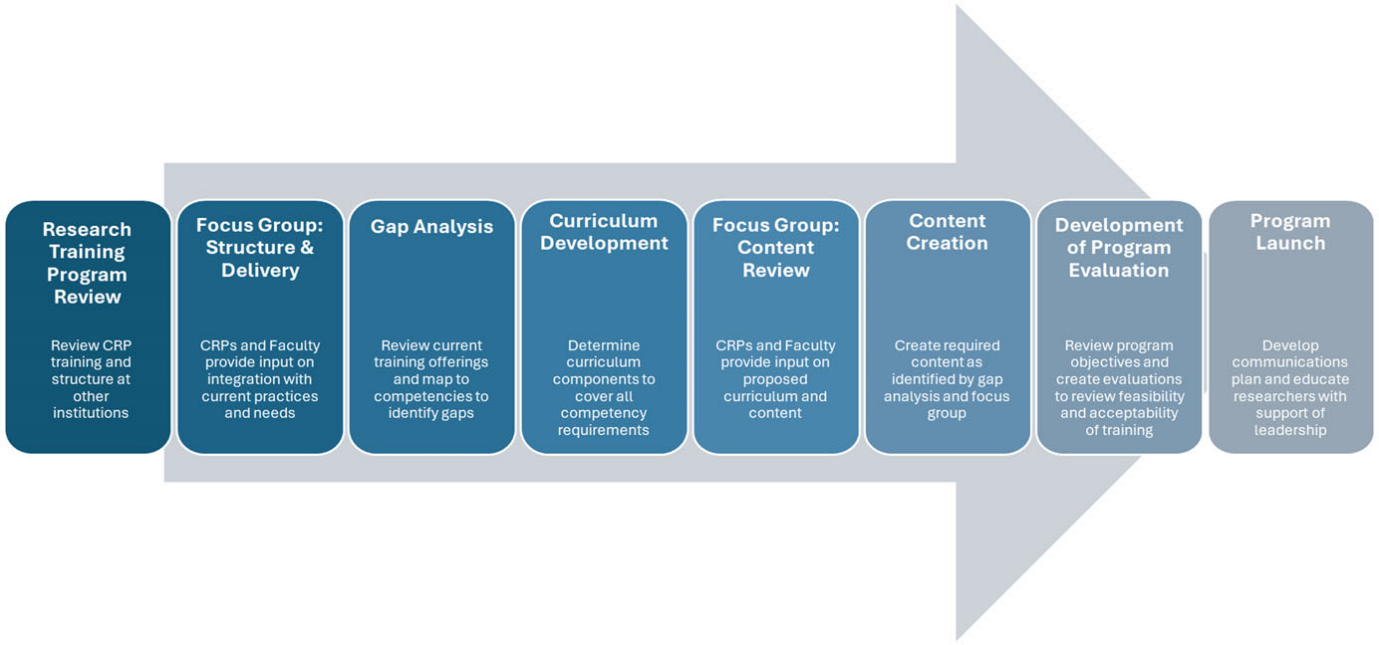
UTSW CRF program development. This figure displays the process of the UTSW CRF program development, from the research training program review to UTSW program launch (time frame: approximately 6−8 months). CRF = Clinical Research Foundations; CRP = clinical research professional; UTSW = University of Texas Southwestern.

As a first step, we reviewed clinical research training programs available at other institutions [11,15] to determine best practices (Fig. 1). Next, team representatives met with a focus group of approximately 20 current CRPs (research assistants, coordinators, managers, and nurses) and faculty engaged in clinical research to discuss program structure and delivery. An online self-paced program that had the flexibility to work in conjunction with other onboarding requirements was strongly supported, both by reviews from other institutions and from UTSW’s focus group (Fig. 1). Since the CRF program needed to be available to UTSW affiliate sites, it was hosted in the Collaborative Institutional Training Initiative (CITI) program portal (https://about.citiprogram.org/), which is commonly used across institutions to provide training resources for research ethics, compliance, and Good Clinical Practice. Utilization of this existing training portal provided ease of access and a well-established reporting structure that enabled monitoring of course completion. While developing the program, it became clear that existing CRPs and faculty had interest in the content, so a video library was developed and hosted on an external website for those wishing to access the content on an *ad hoc* basis. This library contains all locally produced content and is widely linked through UTSW websites to direct researchers to the modules.

### Curriculum development

The CRF program was designed to orient staff at UTSW and affiliate centers; therefore, a collaborative approach across the institution was required to create an effective training program. The curriculum development team was comprised of PhD researchers, current and ex-coordinators, regulatory experts, and faculty, who provided input and content for different program components. Program development took around 6 − 8 months from inception to launch.

To ensure the program covered core clinical research skills, we utilized the JTF CRP competency domain framework [8]. The JTF framework contains eight domains: 1) Scientific Concepts and Research Design; 2) Ethical and Participant Safety Considerations; 3) Investigational Products Development and Regulation; 4) Clinical Study Operations – Good Clinical Practice; 5) Study and Site Management; 6) Data Management and Informatics; 7) Leadership and Professionalism; and 8) Communications and Teamwork. Each of these eight domains contain competency statements with leveled objectives at Fundamental, Skilled, and Advanced Levels [9]. Since this was a foundational program designed for new research staff at point of hire, we used the Fundamental Level objectives for each of the JTF competencies and designed our curriculum and program components to cover all eight clinical research domains and statements accordingly (Table 1).

**Table 1.** CRF curriculum, learning objectives, and competency mapping

Module 1. Scientific Principles of Clinical Research		
Goal: Learners will be able to differentiate medical care from clinical research, define different types of research and the objectives of each, and discuss why it is important to disseminate research results.		
**Unit**	**Learning Objectives**	JTF Competency Domains*
Principles in Research (Length 35:51 min)	Define clinical research, delineate trial phases, and explain key concepts of research.	1, 8
Key Concepts of Clinical Research (Length 35:26 min)	Understand the science of clinical research, identify research hypotheses, and outline experiment plans.	1, 8
Methods of Research (Part 1) (Length 26:33 min)	Identify observational studies and understand their operations.	1, 4, 8
Methods of Research (Part 2) (Length 26:02 min)	Identify interventional study designs, understand their operations, and identify the components of a research publication.	1, 4, 8

Module 2. Introduction to Clinical Research at UTSW Medical Center and Affiliates		
Goal: Introduce clinical research professionals to the Offices and Committees that interface with Clinical Research at UTSW and Affiliate Hospitals and outline their roles.		
**Unit**	**Learning Objectives**	Competency Domains*
Leadership Welcome and Departments (Length 23:15 min)	Understand the research structure at UTSW Medical Center.	7, 8
Research Committees (Length 32:18 min)	Identify and understand committees involved in clinical research approvals.	4, 5, 7
Other Approvals and Resource Offices (Length 21:44 min)	Outline and understand other approvals and offices involved in clinical research.	4, 5, 7
Performance Site Approvals (Length 20:01 min)	Recognize and understand the role, importance, and process required for performance site approvals.	4, 5, 7
Resources (Length 34:50 min)	Identify and understand institutional resources available to support clinical research.	7, 8

Module 3. CITI Clinical Research Elements		
Goal: Provide clinical research professionals with basic and thorough training tailored to the researcher’s critical role in the conduct of clinical trials.		
**Unit**	**Learning Objectives**	Competency Domains*
Clinical Research Coordinator Responsibilities (ID 16576)	Appreciate maintenance of study documentation and binders; review the informed consent process and understand study closeout procedures.	2, 4, 5, 7
Funding, Financial Management, and Budgeting (ID 16752)	Differentiate types of funding, pre- and post-award processes, clinical trial agreements and coverage analysis; distinguish routine versus research costs.	2, 5
Informed Consent (ID 16758)	Understand US Department of Health and Human Services and Food and Drug Administration regulations, International Council for Harmonization and Good Clinical Practice guidelines; review informed consent forms, recruitment materials, and study team responsibilities.	2,3
Planning Research (ID 16751)	Explore the history of clinical trials, and drug and device development; appreciate the design of clinical trials and understand their phases; identify study sponsors, review protocol components, feasibility, and study team roles and responsibilities.	1, 2, 3, 4, 5
Principal Investigator Responsibilities (ID 16755)	Understand principal investigator responsibilities, adverse event reporting, site visits and monitoring, Food and Drug Administration/Institutional Review Board inspections, and the authorship and publication process	3, 4, 5, 8
Protocol Review and Approvals (ID 16754)	Identify and understand other committees such as Data Safety and Monitoring Boards, radiation and Institutional Biosafety Committees, and investigational drug services.	4
Site Management, Quality Assurance, and Public Information (ID 16759)	Understand lab storage, records retention, point-of-care testing, research integrity, and the maintenance of confidentiality.	4, 7
Sponsor Responsibilities (ID 16757)	Recognize sponsor IND/IDE responsibilities, sponsor delegation of authority documents, sponsor monitoring, and reporting for sponsor risk and safety.	3, 4, 8
Working with the Institutional Review Board (ID 16753)	Appreciate the purpose of the Institutional Review Board, types of review and required documents, reportable events, compliance, deviations, safety monitoring, and study closeout processes.	4
Project Management for Clinical Trials (ID 17864)	Explore project management principles, design processes, and workflows; understand team leadership, risk management, and effective communication.	2, 3, 5, 7, 8
Preventing and Identifying Misconduct and Noncompliance (ID 17865)	Define research terminology, detect and prevent research misconduct and fraud, understand data integrity, and develop Corrective and Preventive Action plans.	4, 7
Training and Mentoring (ID 17866)	Understand the Clinical Research Coordinator onboarding process and effective mentoring strategies.	8
Financial Management of Clinical Trials (ID 17867)	Identify research costs, understand subject reimbursement, compliance with regulations, prevention of false claims and basic budget review.	5
Subject Recruitment and Retention (ID 17868)	Understand recruitment regulations, planning, timelines and enrollment goals, effective recruitment techniques, cultural competency and barriers, and identify vulnerable populations.	2, 5, 7
Statistics and Data Management of Clinical Trials (ID 17869)	Identify basic statistical methods; define sample size, clinical trial statistics, randomization and blinding, interim analysis, data management, and risk-based data monitoring.	4, 6
Specialty Areas and Regulatory Requirements (ID 17870)	Recognize electronic systems and signatures and understand the shipping and receiving of hazardous goods.	1, 3
Length of CITI module completion varies dependent on the familiarity and knowledge that the learner has of the topic, and the overall length and amount of content in the module itself. Generally, modules are designed to each take around 30−45 minutes to complete.		

*Information about the Competency Domains can be found within the text of this article and in [8,9]. Abbreviations: CITI = Collaborative Institutional Training Initiative; CRF = Clinical Research Foundations; IDE = investigational device exemption; IND = investigational new drug; UTSW = University of Texas Southwestern.

We initially performed a gap analysis, mapping the CITI clinical research coordinator comprehensive course training modules to the relevant JTF domains and leveled objectives (Fig 1, Table 1) in order to determine the content that needed to be created by the UTSW team. Our review of other clinical research training programs combined with the work from our focus group helped us to decide that a combination of existing online CITI training modules and UTSW-created material would provide optimal balance between generalized clinical research education and institutionally tailored content. Next, we reviewed our proposed content with subject-matter experts and focus groups to ensure it was relevant, manageable for researchers to complete during their onboarding period, and accessible for those who may be taking *ad hoc* courses outside of the mandatory requirement (Fig. 1). We decided the curriculum would consist of three major modules: 1) Module 1, Scientific Principles of Clinical Research; 2) Module 2, Introduction to Clinical Research at UTSW Medical Center and Affiliates; and 3) Module 3, CITI Clinical Research Elements (Table 1). The UTSW-produced Modules 1 and 2 contain tailored content, which not only fulfills the relevant JTF competency domains [8] but also relays institution-specific information for new research staff. The CITI courses in Module 3 provide a more generalized training in clinical research skills and Good Clinical Practice, adhering to the JTF matrix (Table 1). Efforts were centralized through the UTSW OCR, Human Research Protection Program, and the Clinical and Translational Science Award Program, with content and contributions provided from subject-matter experts across more than 30 offices and departments at UTSW Medical Center and affiliates. All major areas involved in clinical research at UTSW were invited to provide a short video (4−5 minutes or less) outlining their role and providing contact details for researchers who may wish to learn more or had specific questions. In addition, experienced clinical research faculty contributed to the creation of Module 1 around the science behind clinical research. Full details of all contributors can be found in the Acknowledgments.

When completed through the CITI portal, all modules contained a quiz component at the end of each series to assess understanding; a score of at least 80% is required to advance to the next module. A course certificate is provided for individuals who complete the entire course through the CITI training portal. A manual titled the “Clinical Research Handbook” was developed to accompany the CRF course and provides more than 250 pages of complementary information. The manual acted as a reference guide for CRPs if they needed more information on a particular topic or wanted to revisit a certain area of training.

### Program evaluation

To evaluate the CRF program, we established a two-pronged approach, gathering both quantitative and qualitative data to review success and aid continuous quality improvement. Anonymous evaluations were sent to all individuals who completed the full course through the CITI portal using the Research Electronic Data Capture system (REDCap; Supplemental Material 2) [17]. To assess our population, respondents were asked whether they were assigned the CRF training as part of mandatory onboarding and if they had previous research experience. Quantitative measures included questions about improvements in clinical research knowledge, understanding, preparedness, and institutional awareness, which participants rated on a 5-point Likert scale of agreement to assess acceptability and course perception. Individuals were also asked about the value of resources and whether training requirements were realistic and feasible (Supplemental Material 2). Usefulness, feasibility, and acceptability of the program were determined as scores of either 4 or 5 on the 5-point Likert scale. Quoted percentages are calculated from raw data. Qualitative feedback was elicited through free-text options, allowing participants to provide feedback on the curriculum and areas potentially in need of improvement.

### Institutional commitment

To ensure staff had adequate time to complete the training, extensive communications from the OCR informed principal investigators and research managers about the new training requirement. Additionally, institutional leaders underlined the importance of CRP career development to principal investigators and managers (Fig. 1). Centralization of program administration through the OCR was instrumental in ensuring adherence to requirements and underscoring the institutional commitment to this program.

## Results

### CRF program feasibility

The UTSW CRF training program was launched in July 2023. In the first 10 months of availability, 427 individuals completed the full program through the CITI training portal. Most individuals who completed the training were from UTSW Medical Center (89%), although participants from affiliate sites, including Children’s Health (3%), Parkland Health (1%), and Texas Health Resources (3%), also completed the CRF training course (Table 2).

**Table 2.** Characteristics and details of CRF training program completers (July 2023−May 2024)

	# Number	%
**CRF training completers**		
Total	427	–
# Required to complete CRF training as part of onboarding	104	24%
# Not required to complete CRF training	323	76%
**CRF completers by primary institution**		
UTSW Medical Center	378	89%
Children’s Health	12	3%
Parkland Health	6	1%
Texas Health Resources	12	3%
Texas Scottish Rite Hospital for Children	1	<1%
Other	18	4%
UTSW CRF completers by job title*		
Clinical Research Assistant (I and II)†	59	20%
Clinical Research Coordinator (I, II, and lead)†	35	12%
Assistant/Associate Professor	27	9%
Student Assistant/Worker	20	7%
Clinical Research Project/Manager/Supervisor†	18	6%
Research Assistant/Technician (I and II)	17	6%
Residents	17	6%
Research RN†	15	5%
Clinical Fellow	11	4%
Registry Assistant/Associate/Specialist	10	3%
Research Associate	7	2%
Program Coordinator	6	2%
Temporary Staff	6	2%
Postdoctoral Researcher	5	2%
Postdoctoral Trainee Clinical	5	2%
Assistant Instructor	4	1%
Other	31	11%

*0f the 378 UTSW completers, 85 were no longer at UTSW at the time of data analysis; therefore, the percentages are based on the 293 existing employees.

†Job titles required to complete CRF training as part of mandatory onboarding practices. CRF = Clinical Research Foundations; RN = registered nurse; UTSW = University of Texas Southwestern.

Of those that completed the course, 43% held a CRP job title, as determined by our Human Resources review (Table 2), and these included learners who were required to take the CRF training as part of mandatory onboarding. Interestingly, individuals with other job titles (57%) also completed the CRF training, as outlined in Table 2. Of the 427 completers, 24% were assigned the training as part of their institutional mandatory onboarding education, while 76% of completers were not required to take the training (Table 2). Unexpectedly, 9% of learners were faculty members and 10% were fellows or residents (Table 2). As of May 2024, the UTSW CRF course has 100% compliance (104/104) for individuals required to complete the training within 30 days of hire as part of their mandatory onboarding education.

### CRF survey completers

Those who completed the CRF training program through the CITI portal were sent an anonymous program evaluation questionnaire; 55/427 participants started the survey (13% response rate) and 49/55 completed all questions (11% incompletion rate). Seventy-one percent (*n* = 35) were required to take the training as part of mandatory onboarding (indicating that they were new to research at UTSW), and 29% (*n* = 14) were existing clinical research staff at UTSW or affiliates. When asked about their years of clinical research experience, 49% of the new hires had no clinical research experience and 34% had 2 years or less. Conversely, for existing research staff, the majority (64%) had 5 or more years of clinical research experience, and their primary reason for enrolling in the CRF training program was because they were “*curious about the course*” (57%).

### CRF program acceptability

**Figure 2.**
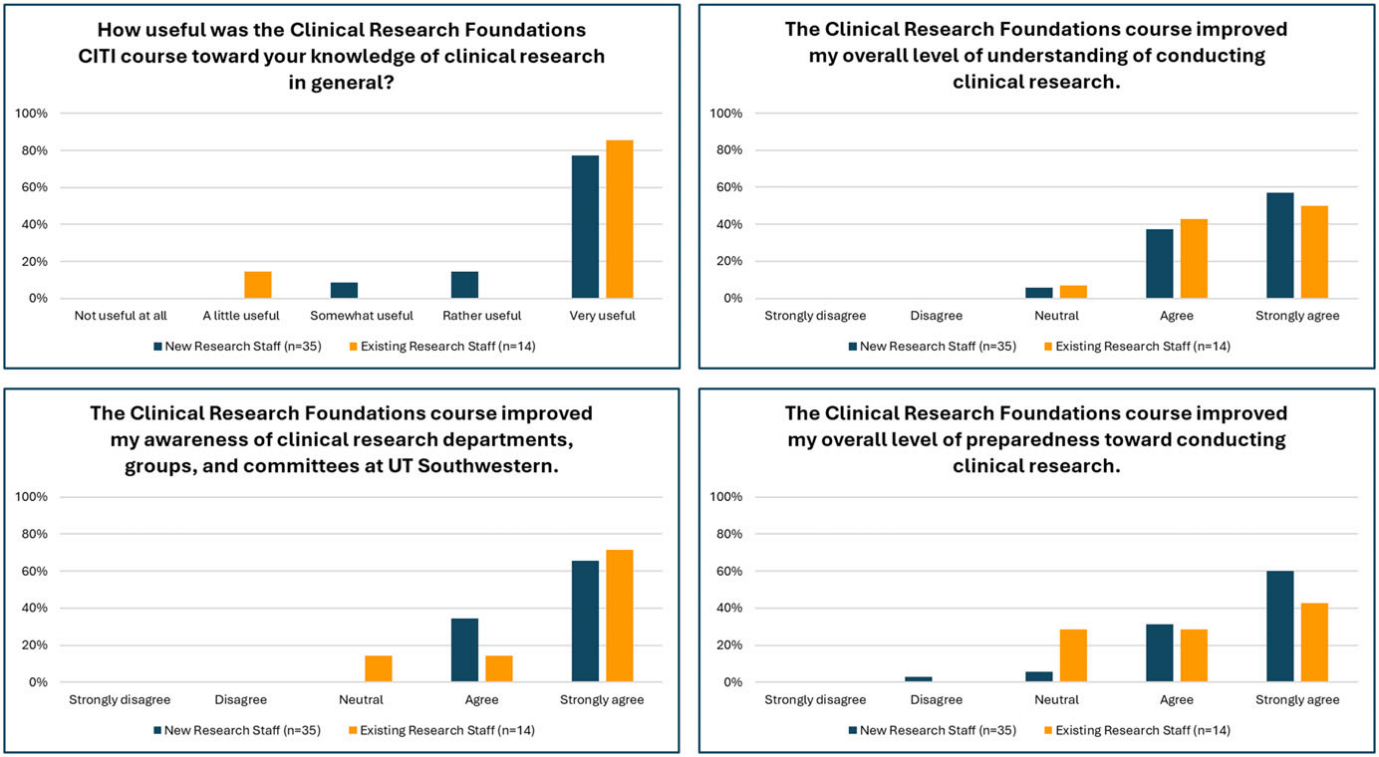
Participant feedback on UTSW training program. Individuals who completed the CRF training program reported useful clinical research knowledge and improvements in understanding, awareness, and preparedness for clinical research conduct at UTSW Medical Center. CRF = Clinical Research Foundations; UTSW = University of Texas Southwestern.

Overall, learners indicated that they overwhelmingly found the UTSW CRF training either useful or very useful in contributing to their knowledge of clinical research (90%), with existing research staff indicating a similar level of usefulness when compared to new clinical research staff (86% vs. 91%, respectively) (Fig. 2). Ninety-four percent of participants (both new and existing) also agreed or strongly agreed that the CRF course improved their overall understanding of clinical research conduct. This was true for both new hires (94%) and existing (93%) employees (Fig. 2), indicating that this training was acceptable for research staff with various levels of experience.

The training was designed not only to include modules outlining general clinical research principles but also to provide tailored guidance on the conduct of clinical research at UTSW and affiliated sites. Ninety-six percent of all individuals indicated that they agreed or strongly agreed that the course improved their awareness of clinical research departments, groups, and committees at UTSW. Institutional content (modules about clinical research departments, groups, and committees at UTSW) was well received by all new research staff (100%) and the majority of existing research staff members (86%) (Fig. 2). Overall, participants agreed or strongly agreed that the CRF training course improved their general level of preparedness toward conducting clinical research (both new and existing: 86%), with 91% of new clinical researchers reporting feeling more prepared because of this education (Fig. 2).

**Figure 3.**
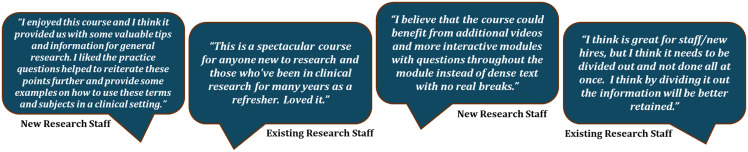
Participant feedback on UTSW training program. Individuals who completed the CRF training program broadly commented that they felt the course was beneficial with useful content although it contains a lot of information and may benefit from some interactive components. CRF = Clinical Research Foundations; UTSW = University of Texas Southwestern.

In addition to the quantitative data, participants were asked to comment on the strengths and weaknesses of the CRF training program. Broadly, learners enjoyed the program and felt that it was beneficial for both new and existing CRPs, with useful information and resources that could be accessed throughout their clinical research journey (Fig. 3). Nevertheless, participants indicated that since the course covers a large volume of information, it may be beneficial to include some interactive components or to divide the information into smaller sections to prevent learners from feeling overwhelmed (Fig. 3).

## Discussion

Our results demonstrate that the UTSW CRF program is feasible and acceptable with a high rate of uptake and satisfaction on an institutional scale. Participants completing the program noted feeling more adequately prepared for their clinical research roles. Our data indicate that designing and implementing a required institutional training program for CRPs is feasible and acceptable. Key contributors to feasibility included an institutional commitment to increasing CRP professionalism, as well as partnering with key leaders in Human Resources, academic departments, and research centers [2,4,6]. By making the CRF training program a mandatory onboarding requirement for all new clinical research staff, the institution ensured that all new research staff are provided with a fundamental grounding in clinical research conduct at UTSW. To date, not all academic medical centers have committed to mandatory training requirements; however, we found that using this strategy alongside appropriate escalation strategies (when needed) has ensured 100% completion rates.

Even though the UTSW CRF training program was based on the Fundamental Level of the JTF competencies, it unexpectedly had substantial uptake among experienced researchers on campus, including faculty and students (Table 2) [8,9]. Despite the high level of experience of some of these individuals, they reported improved institutional understanding and clinical research knowledge (Fig. 2). These findings led the Harold C. Simmons Comprehensive Cancer Center, one of the largest centers at UTSW, to require completion of the CRF training program for all clinical research staff, regardless of years of experience, to ensure standard foundational knowledge. As a result, we made Module 1 (Scientific Principles of Clinical Research) and Module 2 (Introduction to Clinical Research at UTSW Medical Center and Affiliates) available on our public website, thereby accessible to all researchers as a resource library. Furthermore, 19% of the individuals that completed the program are faculty, fellows, or residents, which demonstrates the versatility of the CRF training program. Such results were not found when compared to other CRP programs [13], so the utility of this particular curriculum design for clinical- and research-related roles is noteworthy.

A novel aspect of the CRF training program was the integrated approach to learning, with utilization of both UTSW-produced training content alongside commercially available CITI Clinical Research Modules. To date, most CRP curriculums have been created solely by the institution, which makes them more challenging to recreate or modify, whereas Modules 1 and 3 of the CRF curriculum are broadly applicable and could be utilized by any clinical research training program. Additionally, such programs are predominantly instructor-led, thereby adding to the need for teaching-related salary support [10 − 12,15]. The self-paced, online nature of our program allows flexibility in training completion. Such versatility has resulted in increased levels of support from principal investigators and managers, since the training does not negatively impact existing department- or study-specific onboarding processes. Furthermore, UTSW CRF’s commercially available online modules reduce the cost of staff time for both course development and delivery, resulting in an economically effective model. By leveraging both the CITI courses and platform for hosting the training, we have created a pliable plug-and-play program, where modifications can easily be made to tailor courses according to audience and institutional needs and permitting similar programs to be created at other research centers (Fig. 1, Table 1). These shareable resources provide opportunities for institutions to create their own tailored training with minimal effort and cost, with only CRF Module 2 needing modification for alternative institutional needs. Given the consequent opportunities for modification and dissemination, we are working with academic and health institutions across the Texas Regional Clinical and Translational Science Award Consortium to create versions of this program for diverse audiences (e.g., faculty, trainees, community members) and institutions.

The online program provides flexibility for learners, yet clinical research naturally contains elements which lend themselves to interactive activities or practical observation. Therefore, we plan to develop in-person activities based on practical clinical research skills (e.g., informed consent), which will accommodate different learning styles and offer variety to the available learning modalities. As the CRF program continues to grow at UTSW and its affiliated centers, supplementing the current curriculum with a complimentary mentoring program may prove a promising strategy. Such a complementary program would foster continued professional development, which would promote increased wellbeing and CRP retention [4,6,18]. Plans are underway to incorporate this component into our current training structure.

In conclusion, delivery of the UTSW CRF training program has proven feasible, with high levels of acceptability for new and existing CRPs at UTSW Medical Center. The CRF program continues to be an integral part of institutional clinical research training. Uptake of the curriculum extended far beyond our expected audience, encompassing faculty, clinicians, and trainees; these findings highlight the importance of centralized institutional clinical research training at all levels. By employing a leveled, competency-based framework for program design, we have not only established a well-rounded curriculum for researchers but also provided an opportunity for future growth through the Skilled and Advanced competency levels. Combining CITI courses with institutionally created content allows for a uniquely malleable plug-and-play instructional model for standardized clinical research training that can be modified according to the specific needs of the interested audience or institution.

## Supporting information

Palmer et al. supplementary material Palmer et al. supplementary material

## Supplementary material

The supplementary material for this article can be found at https://doi.org/10.1017/cts.2024.690.
