# Supplementary material for: Completion of a standardizable competency-based research training program improves understanding and preparedness for both new and experienced clinical research professionals
Source: J Clin Transl Sci. 2024 Dec 26;9(1):e19. doi: 10.1017/cts.2024.690 (PMC11795864; doi:10.1017/cts.2024.690)
Supplement: Palmer et al. supplementary material [file S2059866124006903sup001.pdf]

**Supplemental Material 1. List of clinical research job titles at UTSW required to take CRF Program as part of mandatory onboarding training.**

| **Title** |
|---|
| CLIN RESCH ASSIST I |
| CLIN RESCH ASSIST II |
| CLIN RESCH ASSIST LEAD |
| CLIN RESCH COORD I |
| CLIN RESCH COORD II |
| CLIN RESCH COORD LEAD |
| SUPV CLIN RESCH |
| MGR CLIN RESCH |
| MGR RESCH PRGMS |
| PROJECT MANAGER CLINICAL RESEARCH |
| RESCH RN |
| RESCH RN SR |
| SUPERVISOR RESEARCH NURSE |
| MANAGER RESEARCH NURSE |
| ADVANCED PRACTICE RESEARCH NURSE |

**Supplemental Material 2. Research Electronic Data Capture (REDCap) system post-course evaluation survey**

# Survey for Cohort 2: Completed CRF CITI Course

Purpose of this voluntary survey: To gather preliminary insight on the new Clinical Research Foundations training curriculum. The survey should take no more than 2 minutes to complete and is anonymous (unless you provide us with your contact information).Who is being asked to participate: All individuals who have taken the Clinical Research Foundations course in CITI.We thank you all for your support of clinical research training at UT Southwestern Medical Center and we look forward to receiving your feedback!This program was created with support from the Human Research Protections Program (HRPP), Office of Clinical Research (OCR), and the Clinical and Translational Science Award (CTSA) Program.

---

Where do you primarily perform your job (i.e., which hospital)?

- ◯ UT Southwestern
- ◯ Children's Health
- ◯ Parkland
- ◯ Scottish Rite
- ◯ Texas Health
- ◯ Other

---

Since you have selected "Other," please enter where you primarily do research:

__________________________________

---

Are you a new hire OR newly transferred to a clinical research job?
(NOTE: newly hired = someone who was newly hired to the institution within the past few months. Transfer = transitioned roles from a non-clinical research role to a clinical research role)

- ◯ Yes
- ◯ No

---

You have selected "No" to being a new hire or a newly transferred to a clinical research job. Since these are the two categories required to take the Clinical Research Foundations course, please select the reason why you took this course:

- ◯ Manager required
- ◯ Department required
- ◯ Institution required
- ◯ Curious about the course
- ◯ Other

---

You have selected "Other" as the reason for taking this course. Please explain why you took it:

__________________________________

---

**This first series of questions is focused on your background history with clinical research, including training and work experience.**

---

Have you had any previous training in clinical research before taking the Clinical Research Foundations CITI course?

- ◯ Yes
- ◯ No

---

Please check off all of the types of clinical research training you received in the past (not including the Clinical Research Foundations training that you just completed). The training could have been conducted by any department, as long as the goal was to teach about some aspect of clinical research in theory or in practice.

- ☐ Shadowing other staff
- ☐ Mentor
- ☐ Skills check off list
- ☐ In-person training session(s)s on campus
- ☐ Online session(s)
- ☐ Conference (either once or multiple)
- ☐ Offsite training by an outside vendor
- ☐ Other

---

Since you have selected "Other," what other types of research training have you received?

__________________________________

---

Have you had any previous experience working in clinical research before taking the Clinical Research Foundations CITI course?

- ◯ Yes
- ◯ No

How many years of working experience in clinical research did you have in the past?

- ◯ Less than 1 year
- ◯ 1 - 2 years
- ◯ 3 - 4 years
- ◯ 5+ years

**This last series of questions are focused on the new Clinical Research Foundations training curriculum, including the CITI course and website.**

If you need to review the UTSW Clinical Research Foundations website, please click here.

How useful was the Clinical Research Foundations CITI course toward your knowledge of clinical research in general?

- ◯ Very useful
- ◯ Rather useful
- ◯ Somewhat useful
- ◯ A little useful
- ◯ Not useful at all

The Clinical Research Foundations course improved my overall level of understanding of conducting clinical research.

- ◯ Strongly Agree
- ◯ Agree
- ◯ Neutral
- ◯ Disagree
- ◯ Strongly Disagree

The Clinical Research Foundations course improved my overall level of preparedness toward conducting clinical research.

- ◯ Strongly Agree
- ◯ Agree
- ◯ Neutral
- ◯ Disagree
- ◯ Strongly Disagree

The Clinical Research Foundations course improved my awareness of clinical research departments, groups, and committees at UT Southwestern.

- ◯ Strongly Agree
- ◯ Agree
- ◯ Neutral
- ◯ Disagree
- ◯ Strongly Disagree

The Clinical Research Foundations Training website fully and concisely explains the value of this training program.

- ◯ Strongly Agree
- ◯ Agree
- ◯ Neutral
- ◯ Disagree
- ◯ Strongly Disagree

The Clinical Research Foundations training curriculum requirements are realistic.

- ◯ Strongly Agree
- ◯ Agree
- ◯ Neutral
- ◯ Disagree
- ◯ Strongly Disagree

The most helpful module of the Clinical Research Foundations CITI course is:

- ◯ UTSW Leadership Welcome and Major Departments (ID 21058)
- ◯ UTSW Scientific Principles of Clinical Research (ID 21056)
- ◯ UTSW Introduction to Clinical Research at UT Southwestern Medical Center and Affiliates (ID 21059)
- ◯ CITI Project Management for Clinical Trials (ID 17864)
- ◯ CITI Preventing and Identifying Misconduct and Noncompliance (ID 17865)
- ◯ CITI Training and Mentoring (ID 17866)
- ◯ CITI Financial Management of Clinical Trials (ID 17867)
- ◯ CITI Subject Recruitment and Retention (ID 17868)
- ◯ CITI Statistics and Data Management of Clinical Trials (ID 17869)
- ◯ CITI CRC: Overview (ID 16682)
- ◯ CITI Planning Research (ID 16751)
- ◯ CITI Funding, Financial Management, and Budgeting (ID 16752)
- ◯ CITI Working with the Institutional Review Board (IRB) (ID 16753)
- ◯ CITI Protocol Review and Approvals (ID 16754)
- ◯ CITI Principal Investigator (PI) Responsibilities (ID 16755)
- ◯ CITI Clinical Research Coordinator (CRC) Responsibilities (ID 16756)
- ◯ CITI Sponsor Responsibilities (ID 16757)
- ◯ CITI Informed Consent (ID 16758)
- ◯ CITI Site Management, Quality Assurance, and Public Information (ID 16759)
- ◯ CITI CRC Resources (ID 16774)

Why did you rate this module as the most helpful?

__________________________________

The least helpful module of the Clinical Research Foundations CITI course is:

- ◯ UTSW Leadership Welcome and Major Departments (ID 21058)
- ◯ UTSW Scientific Principles of Clinical Research (ID 21056)
- ◯ UTSW Introduction to Clinical Research at UT Southwestern Medical Center and Affiliates (ID 21059)
- ◯ CITI Project Management for Clinical Trials (ID 17864)
- ◯ CITI Preventing and Identifying Misconduct and Noncompliance (ID 17865)
- ◯ CITI Training and Mentoring (ID 17866)
- ◯ CITI Financial Management of Clinical Trials (ID 17867)
- ◯ CITI Subject Recruitment and Retention (ID 17868)
- ◯ CITI Statistics and Data Management of Clinical Trials (ID 17869)
- ◯ CITI CRC: Overview (ID 16682)
- ◯ CITI Planning Research (ID 16751)
- ◯ CITI Funding, Financial Management, and Budgeting (ID 16752)
- ◯ CITI Working with the Institutional Review Board (IRB) (ID 16753)
- ◯ CITI Protocol Review and Approvals (ID 16754)
- ◯ CITI Principal Investigator (PI) Responsibilities (ID 16755)
- ◯ CITI Clinical Research Coordinator (CRC) Responsibilities (ID 16756)
- ◯ CITI Sponsor Responsibilities (ID 16757)
- ◯ CITI Informed Consent (ID 16758)
- ◯ CITI Site Management, Quality Assurance, and Public Information (ID 16759)
- ◯ CITI CRC Resources (ID 16774)

Why did you rate this module as the least helpful?

__________________________________

On a scale of 1 to 5, with 1 being "poor" to 5 being "excellent," how would you rate your overall experience with this Clinical Research Foundations Training Program?

Poor Neutral Excellent

*(Place a mark on the scale above)*

**Finally, we are interested in any other feedback you may have on this program, including what could be improved related to the Clinical Research Foundations training program at UT Southwestern, or clinical research training in general. Please provide your thoughts below.**

Please enter your ideas and feedback and let us know your thoughts! If you want to be contacted, please fill out the "Contact Me!" form at the bottom of this survey. Otherwise, your survey results will remain anonymous.

Thank you for your participation!

Contact Me!

Name

__________________________________

Email

__________________________________

Department

__________________________________
